# Influenza A(H3N2) virus exhibiting reduced susceptibility to baloxavir due to a polymerase acidic subunit I38T substitution detected from a hospitalised child without prior baloxavir treatment, Japan, January 2019

**DOI:** 10.2807/1560-7917.ES.2019.24.12.1900170

**Published:** 2019-03-21

**Authors:** Emi Takashita, Chiharu Kawakami, Rie Ogawa, Hiroko Morita, Seiichiro Fujisaki, Masayuki Shirakura, Hideka Miura, Kazuya Nakamura, Noriko Kishida, Tomoko Kuwahara, Akira Ota, Hayato Togashi, Ayako Saito, Keiko Mitamura, Takashi Abe, Masataka Ichikawa, Masahiko Yamazaki, Shinji Watanabe, Takato Odagiri

**Affiliations:** 1 Influenza Virus Research Center, National Institute of Infectious Diseases, Tokyo, Japan; 2 Yokohama City Institute of Public Health, Kanagawa, Japan; 3 Saiseikai Yokohamashi Nanbu Hospital, Kanagawa, Japan; 4 Saito Children’s Clinic, Kanagawa, Japan; 5 Eiju General Hospital, Tokyo, Japan; 6 Abe Children’s Clinic, Kanagawa, Japan; 7 Ichikawa Children’s Clinic, Kanagawa, Japan; 8 Zama Children’s Clinic, Kanagawa, Japan

**Keywords:** Influenza virus, cap-dependent endonuclease inhibitor, baloxavir marboxil, baloxavir acid, drug resistance

## Abstract

In January 2019, two influenza A(H3N2) viruses carrying an I38T substitution in the polymerase acidic subunit (PA), which confers reduced susceptibility to baloxavir, were detected from epidemiologically unrelated hospitalised children in Japan. The viruses exhibited reduced susceptibility to baloxavir but were susceptible to neuraminidase inhibitors. Only one of the two children had been treated with baloxavir. An epidemiological analysis suggests possible transmission of the PA I38T mutant A(H3N2) virus among humans.

The cap-dependent endonuclease inhibitor baloxavir marboxil became available in Japan in March 2018 for the treatment of influenza virus infection in patients aged 12 years and older and children younger than 12 years weighing at least 10 kg. Between October 2018 and January 2019, baloxavir was supplied to medical institutions that together serve ca 5.5 million people. In December 2018, we detected influenza A(H3N2) viruses exhibiting reduced susceptibility to baloxavir from baloxavir-treated children aged 6 and 7 years [1]. These viruses possessed an I38T substitution in the polymerase acidic subunit (PA), which confers reduced susceptibility to baloxavir [2]. We subsequently increased nationwide monitoring of the baloxavir susceptibility of circulating influenza viruses, irrespective of antiviral treatment [3].

## Detection of polymerase acidic subunit I38T mutant influenza A(H3N2) viruses from hospitalised children

In January 2019, we isolated two influenza A(H3N2) viruses, A/YOKOHAMA/87/2019 and A/YOKOHAMA/88/2019, from two hospitalised children (Table 1). Prior to hospitalisation and virus isolation, both children had received antiviral treatment against influenza. The primary-school child aged 6 years who was infected with A/YOKOHAMA/87/2019 had been treated with a single oral dose of baloxavir on the day of symptom onset and fever resolved within one day of baloxavir administration. Face oedema had developed 2 days after baloxavir administration, although this patient had no underlying diseases. The child was diagnosed with nephritis and hospitalised. The preschool child aged 5 years who was infected with A/YOKOHAMA/88/2019 had received oseltamivir 3 days after onset of illness, although its clinical benefit is greatest when administered within 48 hours of illness onset. Fever tended to resolve after oseltamivir administration. This child had no underlying diseases but was subsequently hospitalised for pneumothorax and subcutaneous emphysema. No epidemiological link was identified between these patients.

**Table 1:** Influenza A(H3N2) viruses detected from hospitalised children, Japan, January 2019 (n = 2)

GISAID isolate ID	Isolate name	Age in years	Onset of symptoms	Antiviral treatment	Day of hospitalisation	Specimen collection	PA substitution^a^	
							Clinical specimen	Virus isolate
EPI_ISL_341452	A/YOKOHAMA/87/2019	6	19 Jan 2019	19 Jan 2019 baloxavir	21 Jan 2019	25 Jan 2019	I38T/I mix (T: 28%)	I38T
EPI_ISL_341454	A/YOKOHAMA/88/2019	5	25 Jan 2019	28–30 Jan 2019 oseltamivir	31 Jan 2019	31 Jan 2019	I38T	I38T

GISAID: Global Initiative on Sharing All Influenza Data; ID: identity; PA: polymerase acidic subunit.

^a^ For deep sequencing analysis, the mean sequencing depth, threshold used and limit of quantitation used were 14,200, 5% and 2, respectively.

Deep sequencing analysis of the isolates using MiSeq (Illumina, San Diego, California, United States) revealed that A/YOKOHAMA/87/2019 and A/YOKOHAMA/88/2019 possessed the PA I38T substitution. These PA I38T mutant viruses possessed different PA sequences and therefore originated from different sources of infection. PA I38 is highly conserved in influenza A and B viruses [1,2]. The I38T substitution was not detected in the Influenza Research Database (www.fludb.org) including 17,227 PA sequences from A(H3N2) viruses until December 2018 [1] or during surveillance studies of baloxavir susceptibility of influenza viruses in Japan (2017/18 influenza season) and the United States prior to the introduction of baloxavir (2016/17 and 2017/18 seasons) [3,4]. Therefore, previous studies concluded that the PA I38T substitution was a baloxavir treatment-emergent substitution [1,2]. The patient infected with A/YOKOHAMA/87/2019 had been treated with baloxavir, indicating the possible emergence of the PA I38T mutant virus under the selective pressure of this drug. In contrast, the patient infected with A/YOKOHAMA/88/2019 was treated with oseltamivir. Usage of baloxavir increased in this influenza season in Japan and an influenza outbreak occurred in the preschool attended by the 5 year-old before this patient’s symptom onset, suggesting a possible acquisition of the PA I38T mutant virus by human-to-human transmission. 

## Antiviral susceptibilities of the polymerase acidic subunit protein I38T mutant viruses

We determined the susceptibilities of the PA I38T mutant viruses to baloxavir and four neuraminidase (NA) inhibitors approved in Japan: oseltamivir, laninamivir, peramivir and zanamivir (Table 2). Antiviral susceptibilities were determined by using a focus reduction assay and a fluorescent NA inhibition assay with the NA-Fluor Influenza Neuraminidase Assay Kit (Applied Biosystems, Carlsbad, California, United States) as previously described [3]. The hydrolysed active form of baloxavir marboxil (baloxavir acid) was purchased from MedChemexpress (Monmouth Junction, New Jersey, United States). Oseltamivir carboxylate, peramivir, and zanamivir were purchased from Sequoia Research Products (Pangbourne, Reading, United Kingdom), and laninamivir was provided by Daiichi Sankyo Co., Ltd. (Tokyo, Japan). Results are expressed as the 50% inhibitory concentration (IC_50_) values, which were calculated by using MikroWin 2000 software (Mikrotek Laborsysteme GmbH, Overath, Germany). To interpret the NA inhibitor susceptibility, the World Health Organization (WHO) criteria based on the fold change of IC_50_ values compared with reference IC_50_ values were applied [5]. These define inhibition of influenza A viruses as normal (< 10-fold increase), reduced (10–100-fold increase) or highly reduced (> 100-fold increase).

**Table 2:** Susceptibility of influenza A(H3N2) viruses detected from hospitalised children, Japan, January 2019 (n = 2)

Isolate name	PA substitution	IC_50_, nM				
		Baloxavir^a^	Neuraminidase inhibitors (WHO criteria^b^)			
			Oseltamivir^a^	Peramivir^a^	Zanamivir^a^	Laninamivir^a^
A/YOKOHAMA/87/2019	I38T	157.87	0.52 (NI)	0.16 (NI)	1.04 (NI)	1.12 (NI)
A/YOKOHAMA/88/2019	I38T	218.89	0.44 (NI)	0.13 (NI)	1.07 (NI)	1.13 (NI)

IC_50_: 50% inhibitory concentration; PA: polymerase acidic subunit; WHO: World Health Organization.

^a^ The median IC_50_ values of A(H3N2) viruses isolated in the 2018/19 influenza season in Japan to baloxavir (n = 22 viruses without the PA I38T substitution) and to oseltamivir, peramivir, zanamivir and laninamivir (n = 69) were 3.22 ± 2.93, 0.22 ± 0.15, 0.10 ± 0.03, 0.48 ± 0.27 and 0.92 ± 0.24, respectively.

^b^ NI: normal inhibition.

The IC_50_ values of the viruses to baloxavir and the NA inhibitors are shown in Table 2. Both PA I38T mutant viruses showed normal inhibition with all four NA inhibitors, but exhibited 49- and 68-fold higher IC_50_ values to baloxavir compared with the median IC_50_ value of A(H3N2) viruses isolated in the 2018/19 influenza season in Japan. No amino acid substitutions associated with reduced susceptibility to NA inhibitors were detected in either virus. These results indicate that the PA I38T mutant viruses had reduced susceptibility to baloxavir, but remained susceptible to NA inhibitors.

## Discussion

In this study, we detected two PA I38T mutant A(H3N2) viruses respectively from two hospitalised children. In addition, during our nationwide monitoring, we detected nine PA I38T or I38M mutant A(H3N2) viruses from baloxavir-treated patients (Table 3). All of these viruses were isolated in humanised MDCK cells, hCK cells, which express high levels of α2, 6-sialoglycans and very low levels of α2, 3-sialoglycans [6]. Deep sequencing analysis revealed that eight of these viruses possessed mixed PA I38T/I or I38T/M/I substitutions in the clinical specimens and six of these eight possessed increased proportion of the PA I38T or I38M substitution after virus isolation. A previous study reported that influenza A/Victoria/3/75(H3N2) viruses with the PA I38T, I38M, or I38F substitutions showed less growth capability than the wild-type virus in cell culture [2]. In contrast, our results indicate that recently circulating A(H3N2) viruses with the PA I38T or I38M substitution grow well, at least in cell culture. 

**Table 3:** Influenza A(H3N2) viruses with the PA I38 substitution detected in the 2018/19 influenza season, Japan (n = 9)

GISAID isolate ID	Isolate name	Age in years	Antiviral treatment	Specimen collection	PA substitution^a^	
					Clinical specimen	Virus isolate
EPI_ISL_332908	A/YOKOHAMA/133/2018	6	3 Dec 2018 Baloxavir	6 Dec 2018	I38T	I38T
EPI_ISL_332910	A/YOKOHAMA/135/2018	7	4 Dec 2018 Baloxavir	7 Dec 2018	I38T/I mix (T: 80%)	I38T
EPI_ISL_340687	A/KANAGAWA/IC1807/2018	14	17 Dec 2018 Baloxavir	20 Dec 2018	I38T/I mix (T: 10%)	I38T
EPI_ISL_337453	A/KANAGAWA/AC1817/2018	8	21 Dec 2018 Baloxavir	25 Dec 2018	I38T/M/I mix (T: 48%; M: 20%)	I38T/M/I mix (T: 40%; M: 21%)
EPI_ISL_340692	A/KANAGAWA/IC1817/2019	9	5 Jan 2019 Baloxavir	8 Jan 2019	I38M/I mix (M: 8%)	I38M/I mix (M: 62%)
EPI_ISL_337460	A/KANAGAWA/IC1827/2019	5	9 Jan 2019 Baloxavir	12 Jan 2019	I38T/I mix (T: 16%)	I38T/I mix (T: 84%)
EPI_ISL_340690	A/KANAGAWA/AC1829/2019	4	12 Jan 2019 Baloxavir	15 Jan 2019	I38T/I mix (T: 18%)	I38T/I mix (T: 16%)
EPI_ISL_340695	A/YOKOHAMA/56/2019	1	11 Jan 2019 Baloxavir	15 Jan 2019	I38T	I38T
EPI_ISL_340699	A/YOKOHAMA/61/2019	4	21 Jan 2019 Baloxavir	25 Jan 2019	I38T/I mix (T: 65%)	I38T/I mix (T: 88%)

GISAID: Global Initiative on Sharing All Influenza Data; ID: identity; PA: polymerase acidic subunit.

^a^ For deep sequencing analysis, the mean sequencing depth, threshold used and limit of quantitation used were 14,200, 5% and 2, respectively.

Of the two children described as infected with a PA I38T mutant virus in this report, one (infected with A/YOKOHAMA/88/2019) had not received baloxavir treatment. Because an influenza outbreak, with possible use of baloxavir, had occurred in this child’s preschool prior to their symptom onset, it could be that the child acquired the mutant virus there. This might indicate that the recently circulating A(H3N2) viruses with the PA I38T substitution have, to some extent, retained replication and possible transmission capability in humans. In this respect, the parents, and a sibling were also diagnosed with influenza 4, 5, or 6 days, respectively, after this child’s symptom onset. Moreover, concerning the second child affected by a mutant virus in this study (i.e. the patient infected with A/YOKOHAMA/87/2019), a sibling of this child was diagnosed with influenza 2 days after the child’s symptom onset. Although we could not obtain specimens from family members of either children, these observations could point to a possible transmission of the PA I38T mutant A(H3N2) viruses among humans.

Among the 11 persons infected with PA I38T or I38M mutant A(H3N2) viruses in the 2018/19 season in Japan, all but one were children younger than 12 years. In Phase III clinical trials of baloxavir marboxil, the PA I38T and I38M substitutions emerged in 36 (9.7%) of 370 A(H3N2) viruses obtained from patients aged 12–64 years and in 18 (23.4%) of 77 A(H3N2) viruses obtained from children aged 6 months to < 12 years [2,7]. Our results confirm that the incidence of the PA I38 mutant viruses in children younger than 12 years is higher than that in patients aged 12–64 years. Baloxavir was approved in the United States in October 2018 for the treatment of acute uncomplicated influenza A and B infections in patients 12 years and older. Since treatment of children younger than 12 years with baloxavir is approved only in Japan at this time, we believe it is important to share our findings.

Koszalka et al. reported that influenza viruses circulating in the Asia-Pacific region between 2012 and 2018 were susceptible to baloxavir [8]. In the United States, the frequency of reduced susceptibility to baloxavir (˃ threefold change) due to a PA I38M substitution was 0.032% for A(H3N2) viruses during the 2016/17 and 2017/18 seasons [4]. In Japan, the frequency of A(H3N2) viruses with any PA I38 substitutions identified as playing a role in baloxavir resistance was 0% in the 2017/18 season; however, it increased between September 2018 and February 2019, with all patients, except one, being treated with baloxavir before specimen collection [9]. Therefore, the baloxavir susceptibility of influenza viruses should be closely monitored.
